# Exploring the perceived impact of social support on the health behaviours of people living with and beyond cancer during the COVID-19 pandemic: a qualitative study

**DOI:** 10.1007/s00520-022-07291-0

**Published:** 2022-07-25

**Authors:** Natalie Miller, Rana Conway, Simon Pini, Caroline Buck, Natalie Gil, Phillippa Lally, Rebecca J. Beeken, Abigail Fisher

**Affiliations:** 1 Department of Behavioural Science and Health, Institute of Epidemiology and Health Care, University College London, 1-19 Torrington Place, Gower Street, London, UK; 2 Leeds Institute of Health Sciences, University of Leeds, Leeds, LS2 9JT UK

**Keywords:** Social support, Health behaviours, Living with and beyond cancer, COVID-19, Qualitative

## Abstract

**Purpose:**

Social support facilitated healthy behaviours in people living with and beyond cancer (LWBC) before the COVID-19 pandemic. Little is known about how social support impacted their health behaviours during the pandemic when social restrictions were imposed. The aim of this study was to qualitatively explore how social support was perceived to impact the health behaviours of people LWBC during the COVID-19 pandemic.

**Methods:**

Semi-structured interviews were conducted via telephone with 24 adults living with and beyond breast, prostate and colorectal cancer. Inductive and deductive framework analysis was used to analyse the data.

**Results:**

Five themes developed. These were (1) Companionship and accountability as motivators for physical activity, (2) Social influences on alcohol consumption, (3) Instrumental support in food practices, (4) Informational support as important for behaviour change and (5) Validation of health behaviours from immediate social networks.

**Conclusion:**

This study described how companionship, social influence, instrumental support, informational support and validation were perceived to impact the health behaviours of people LWBC during the COVID-19 pandemic. Interventions for people LWBC could recommend co-participation in exercise with friends and family; promote the formation of collaborative implementation intentions with family to reduce alcohol consumption; and encourage supportive communication between partners about health behaviours. These interventions would be useful during pandemics and at other times. Government policies to help support clinically extremely vulnerable groups of people LWBC during pandemics should focus on providing access to healthier foods.

**Supplementary Information:**

The online version contains supplementary material available at 10.1007/s00520-022-07291-0.

## Introduction

The number of people living with and beyond cancer (LWBC) in the UK is expected to reach four million by 2030 [1]. People LWBC are at risk of long-term adverse effects of cancer and its treatments, including fatigue, pain, depression, anxiety, fear of cancer recurrence, obesity, hypertension and diabetes [2–5]. However, health behaviours, such as regular physical activity, healthy eating, and low alcohol consumption, as well as maintaining a healthy weight, are associated with improved outcomes [6–9].

The COVID-19 pandemic had negative impacts on the health behaviours of some people LWBC [10–12]. For example, a cross-sectional study of 8730 people LWBC found that the percentage of people who were inactive increased from 4% before to 21% during COVID-19 restrictions [10]. A qualitative study of 18 people LWBC showed that the COVID-19 pandemic negatively impacted their diet and weight [12]. Lower engagement in healthy behaviours in people LWBC during the pandemic is concerning given that engagement was already poor [13]. Many behaviour change theories such as the Social Cognitive Theory, Social-Ecological Model and Self-Determination Theory recognise that social factors such as social support can facilitate behaviour [14–16]. Before the pandemic, observational and interventional studies showed that social support facilitated healthier eating and physical activity in people LWBC, both during and after treatment [17–19]. There is more limited quantitative research exploring the role of social support in the health behaviours of people LWBC during the COVID-19 pandemic. However, observational studies in general population participants found associations between social support and healthier diet, greater physical activity and lower alcohol consumption, even after adjusting for sociodemographic factors [20–22]. Understanding how social support impacts health behaviour can facilitate the design of more effective, feasible, acceptable and sustainable behaviour change interventions [23].

Qualitative research is crucial for understanding people’s lived experiences of social support and how it impacts behaviour [19]. A meta-synthesis of 39 qualitative studies conducted in people LWBC before the COVID-19 pandemic found that recommendations from trusted individuals to perform physical activity (i.e. informational support), encouragement (i.e. emotional support), and participating in physical activity together (i.e. companionship) all facilitated physical activity [19]. However, the social restriction measures implemented during the pandemic changed how people interact with their social support networks. For example, people diagnosed with cancer receiving immunosuppressive treatment were advised to shield and avoid contact with people outside their household [24]. Furthermore, alternative cancer care models using remote delivery were implemented [12]. At the time of writing, only one qualitative study had explored the perceived impact of social support on health behaviours of people LWBC during the pandemic [11]. Despite indicating that social support may be important for facilitating physical activity, the design was limited by using coding of free text data from open-ended questions in a survey of 28 people [11]. Therefore, the aim of this study was to conduct an in-depth exploration of how social support was perceived to impact the health behaviours of people LWBC during the COVID-19 pandemic.

## Methods

### Design

The current study adopted an interpretivist approach, which assumes that reality consists of people’s subjective experiences and acknowledges the role of the researcher in interpreting the meaning attributed to people’s experiences [25]. One-to-one, in-depth, semi-structured interviews were conducted via telephone as these enable researchers to collect open-ended data and explore people’s experiences of a phenomenon [26].

### Participants and recruitment

Participants were recruited from the Advancing Survivorship Cancer Outcomes Trial (ASCOT) [27]. ASCOT participants were recruited from ten NHS Trusts across London, and Essex between 2015 and 2019 (see Table 1 for inclusion/exclusion criteria). In September 2020, an online survey was embedded to assess the impact of the COVID-19 pandemic on health behaviours. At the end of the survey, participants were given the option to consent to in-depth qualitative interviews. Participants were included in the current study even if they had experienced a recurrence or new cancer diagnosis since trial enrolment to maximise sample diversity [28]. Demographic and medical characteristics reported in the COVID-19 questionnaire were used in the current study to contextualise the findings. Ethical approval for the current study was obtained through the National Research Ethics Service Committee South Central—Oxford B (reference number 14/SC/1369).

**Table 1:** Selection criteria for the ASCOT trial

Inclusion criteria	Exclusion criteria
• Adults (aged ≥ 18 years)	• Diagnosed with metastatic cancer
• Diagnosed with non-metastatic breast, prostate or colorectal cancer (stages I–III)	• Receiving active-anti cancer treatment requiring hospital admission
• Not receiving active anti-cancer treatment (except oral anti-cancer treatments taken at home)	• Has severe cognitive impairment
• Can understand spoken and written English	

Of 786 participants who completed the COVID-19 questionnaire, 669 (85%) provided informed consent to participate in interviews. Participants were purposefully selected for this study to capture variation in characteristics expected to influence experiences of social support in health behaviours including age, sex, marital status and living arrangements [28]. Equal numbers of participants in the intervention versus usual care arms were sampled. The target sample size was 16–24, considered to be a sufficient number to achieve meaning saturation: a complete understanding of thematic codes [29].

Participant characteristics are shown in Table 2. The sample consisted of 12 male and 12 female participants living with and beyond breast (*n* = 11; 46%), prostate (*n* = 7; 29%) and colorectal (*n* = 6; 25%) cancer. Their ages ranged from 41 to 87 years (*M* = 61.6, SD = 12.7), and although the majority reported their ethnicity as White British (*n* = 18; 75%), a range of ethnicities were represented. Participants lived with immediate family (including children) (*n* = 14; 58%), partners only (*n* = 4; 17%), friends (*n* = 1; 4%), or alone (*n* = 5; 21%). Interviews lasted from 36 to 113 min (*M* = 62 min).

**Table 2:** Participants’ demographic and medical characteristics (*N* = 24)

	*M* (SD)	Range	*n* (%)
Age (years)	61.6 (12.7)	41–87	
Time since diagnosis (years)^a^	7.95 (0.89)	5.78–9.12	
Sex			
Male			12 (50)
Female			12 (50)
Ethnicity			
White British			18 (75)
White Irish			1 (4.2)
Indian			1 (4.2)
Black Caribbean			1 (4.2)
Pakistani			1 (4.2)
White other			1 (4.2)
Asian other			1 (4.2)
Cancer type			
Breast			11 (45.8)
Prostate			7 (29.2)
Colorectal			6 (25)
Marital status			
Married/living with partner			16 (66.7)
Single			5 (20.8)
Divorced/separated/widowed			3 (12.5)
Living area			
Small town			10 (41.7)
City			6 (25)
Village			5 (20.8)
Large town			3 (12.5)
Living arrangements			
With immediate family (including children)			14 (58.3)
With partner only			4 (16.7)
With friends			1 (4.2)
Alone			5 (20.8)
ASCOT condition			
Usual care group			13 (54.2)
Intervention group			11 (45.8)

*M*, mean; *SD*, standard deviation

^a^This reflects time since initial breast/prostate/colorectal cancer diagnosis. Two participants in the sample had experienced a distant recurrence since the time of their initial diagnosis

### Data collection

Interviews were conducted between May and July 2021. COVID-19 restrictions in the UK during the data collection period are shown in Table 3. All interviews were conducted by NM, a White British female Health Psychology researcher with no prior relationship with study participants. Interviews were guided by a semi-structured interview schedule informed by Social Cognitive Theory [16] (Supplementary Information). All interviews were audio-recorded and transcribed verbatim by a transcription company with UCL data sharing agreement.

**Table 3:** Stages of COVID-19 lockdowns in England, UK (Institute for Government, 2021)

Month	Events
March 2020	First UK lockdown announced
April 2020	Lockdown planned to be extended
May 2020	Government announces a conditional plan for lifting lockdown People who cannot work from home advised to return to workplace but avoid public transport
June 2020	Phased re-opening of schools Non-essential shops re-open Government announces relaxing of social restrictions and 2 m social distancing rule First local lockdown announced in Leicester and parts of Leicestershire
July 2020	First local lockdown commences in Leicester and parts of Leicestershire Pubs, restaurants and hairdressers re-open Local authorities gain additional powers to enforce social distancing
August 2020	Eat Out to Help Out scheme Indoor theatres, bowling alleys and soft play re-open
September 2020	Indoor and outdoor social gatherings of more than six people banned New restrictions announced: return to working from home and 10 pm curfew for hospitality
October 2020	Three-tier system of COVID-19 restrictions commences Second lockdown announced
November 2020	Second lockdown commences
December 2020	Second lockdown ends Return to a three-tier system of restrictions Restrictions relaxed for Christmas Tier 4 alert announced and comes into force in many areas of England
January 2021	Third lockdown commences
February 2021	Government announces roadmap for lifting the lockdown
March 2021	Primary and secondary school students return to school
April 2021	Outdoor pubs, restaurants and non-essential shops re-open
May 2021	Two households of up to six people can meet indoors

### Analysis

Interviews were analysed using framework analysis following the stages outlined by Gale and colleagues [30]. NM deductively coded all transcripts using the four types of social support proposed by Cohen and Wills: emotional, informational, social companionship, and instrumental support [31]. NM also inductively coded the transcripts in accordance with the aims of the study and developed a framework which integrated both pre-determined and data-driven codes. AF double-coded three randomly selected interviews. NM and AF met to discuss any discrepancies and NM refined the framework accordingly. Next, the data was entered into NVivo software version 12 [32], and the amended framework was applied to all transcripts. The data were then summarised in a framework matrix, and interpreted, and themes were identified by exploring relationships between categories. Throughout the analysis process, the research team (NM, AF, RC, SP, CB, NG) met regularly to discuss and refine themes. The transition between initial frameworks to final themes is shown in Supplementary Information.

## Results

Five themes developed from the analysis. Themes and corresponding quotes followed by participants’ sex, age and living arrangements are summarised in Table 4 and context is provided in the results.

**Table 4:** Summary of themes and representative quotes related to how social support impacted the health behaviours of people LWBC during the COVID-19 pandemic

Themes	Examples of quotes
Companionship and accountability as motivators for physical activity	**Opportunity to socialise** “Covid really made people decide to go for walks because they knew it was the only way at the time we could get to socialise and get to see one another.” (Female, 48y, immediate family) **Reduces loneliness** “It’s been quite a lonely time, hasn’t it?… Yes, so just nice to actually be with a person.” (Female, 61y, immediate family) **Opportunity to talk** “When you’re walking and talking, you’re kind of secluded and you can go off into deeper issues.” (Female, 61y, immediate family) **Accountability for people** “If there are two of you, you’re more likely to go and do [a walk] than if you’re on your own. If it starts raining, then you might think oh I don’t really fancy doing that now. There’s nobody to let down except yourself.” (Female, 61y, immediate family*)* **Accountability for dogs** “The dog walking was really the only exercise I was getting.” (Female, 61y, partner) **Perceived lack of need for support in physical activity** “Even if they hadn’t have wanted to go, I would be going for a walk myself anyway.” (Female, 48y, immediate family) **Matching of physical activity abilities** “I prefer going on my own because I can walk at my pace.” (Male, 68y, partner)
Social influences on alcohol consumption	**Influence from family members at home to drink alcohol** “I definitely drank more during the pandemic, especially on the – sharing a bottle of wine, you know, most nights.” (Female, 61y, immediate family) “If you’re just living with somebody then you’ll tend to do the same as them.” (Female, 49y, immediate family) “[My mum] doesn’t drink either… that does help, because… it’s not the main focus of an evening.” (Female, 41y, immediate family) **Lack of social gatherings** “I did used to drink quite a bit too much and whereas a lot of people during lockdown seem to have gone the other way, it helped me because there were no big social gatherings.” (Female, 61y, partner)
Instrumental support in food practices	**Partner support in food practices** “It decides what I’m going to eat for me. I don’t get the choice. I have what she has bought.” (Male, 58y, immediate family) “The cook predominantly is me… I don’t mind cooking… My Mrs can’t cook.” (Male, 45y, immediate family) “[Sharing the food shopping] definitely helps because obviously if one is busy then the other one can still go and get whatever.” (Female, 50y, immediate family) “I do find that a bit of a bugbear that [my partner] doesn’t cook, especially when he was furloughed for seven months, and I’d come in from work and then I’d have to start cooking.” (Female, 61y, partner) **Reliance on friends and neighbours** “Before the systems were set up in position as well as they are now, originally that was always a struggle because we were relying on friends to get us our shopping.” (Female, 41y, immediate family) **Perceived lack of need for support from communities** “I did have volunteers who would have done shopping had I needed but as it turned out I didn’t. I managed to get by on my own quite well.” (Female, 68y, alone) **Government food parcels** “They weren’t concerned whether you were black, white, brown or indifferent. They just sent a hamper… the stuff that I received wasn’t the stuff that I eat… I don’t want to have tins of potatoes or tins of curry. Eww… Give me fresh produce.” (Female, 61y, alone) “[The food parcels] just made you feel like people were looking after you.” (Female, 41y, immediate family)
Informational support as important for behaviour change	**Advice from informal social networks** “[My partner] said about getting an exercise machine, and I looked it up on Amazon and ordered it.” (Male, 63y, immediate family) “It’s like a bit of a double whammy because you know that the person really cares about you. So, you know that every piece of advice they’re giving you is based on… your wellbeing.” (Female, 45y, friends) “If [my partner] implied that I needed to lose some weight then I’d probably be quite offended.” (Female, 61y, immediate family) **Advice from formal social networks** “I got a little book with all the calorific values of various foods in.” (Male, 68y, alone)
Validation of health behaviours from immediate social networks	**Validation of health behaviours** “[My partner] doesn’t object. She lets me get on with [exercising].” (Male, 80y, partner) **Compliments** “[My children] said, “Wow, you can really see how much weight you’ve lost… It’s good because you think oh, yes it’s worth doing, worth carrying on.” (Female, 75y, partner) **Criticisms** “[My partner] said he didn’t want to reach old age and be stuck with… a sort of ageing lush…. that sort of struck home… I don’t want to be a drunk old woman. So yes, the time has come.” (Female, 61y, partner) “My daughters? They think I’m a lazy good-for-nothing… I think they know I’m stubborn, as well. I don’t like being told what to do.” (Male, 68y, immediate family)

1. Companionship and accountability as motivators for physical activity

Most participants described how they went on walks with family and friends during the pandemic and that companionship, rather than health benefits, motivated them to be active. Walking with others provided one of the only opportunities to socialise with non-household members and reduced the loneliness caused by the social restriction measures. Walking with family and friends also meant that people could talk about “deeper issues”, which was partly facilitated by being in a “secluded” outdoor space compared to an indoor space. This opportunity to talk benefitted people’s mental wellbeing. Participants also perceived an obligation to turn up and not let others down. This sense of accountability helped people overcome barriers to physical activity, such as lack of self-motivation and bad weather. Owning a dog also motivated individuals to stay physically active during the pandemic due to a sense of accountability and obligation. However, individuals who were already self-motivated to exercise expressed a lack of need for companionship and were happy to walk alone. Also, individuals who had different walking abilities to their family or friends experienced a mismatch in the support received and desired when walking together. Some felt frustrated by this and preferred walking alone.

Covid really made people decide to go for walks because they knew it was the only way at the time we could get to socialise and get to see one another. (Female, 48y, immediate family)

Although companionship and accountability were important motivators for physical activity, participants also described how the people around them had positive and negative impacts on other health behaviours such as alcohol consumption.

2. Social influences on alcohol consumption

Many participants described how they “drank more” alcohol during the pandemic, due to increased time drinking with family. However, for individuals who did not drink, living with others who also did not drink helped facilitate their lack of drinking, as there was no social norm created to drink at home. Some individuals, particularly those who lived alone or with partners who did not drink, described how the lack of social events during the pandemic helped them reduce their alcohol consumption as there was less temptation to drink.

I definitely drank more during the pandemic, especially on the – sharing a bottle of wine, you know, most nights. (Female, 61y, immediate family)

In addition to social influences having positive and negative effects on alcohol consumption during the pandemic, participants described how receiving instrumental support in food practices had mixed effects on dietary intake.

3. Instrumental support in food practices

Many participants, particularly males living with female partners, described how their partners provided instrumental support by doing all the food shopping and cooking during the pandemic. However, this support often resulted in a perceived loss of agency as individuals experienced less “choice” over what they ate. Individuals whose partners did all the cooking often described how their partner was more interested and competent in cooking than themselves and this arrangement allowed them to gain more nutrition and variety in their diet. In contrast, participants who received no partner support in cooking felt indifferent about this arrangement as they already felt interested and competent in cooking and were thus intrinsically motivated to cook. Some individuals also described how sharing the food shopping and/or cooking with their partner during the pandemic helped both to manage their time better. However, one participant who worked during the pandemic and received no partner support in cooking felt frustrated about the lack of support, especially because their partner was furloughed and had more free time than them.

It decides what I’m going to eat for me. I don’t get the choice. I have what she has bought. (Male, 58y, immediate family)

Participants who were shielding, self-isolating or avoiding supermarkets relied on friends and neighbours to do their food shopping, especially in the early stages of the pandemic when online deliveries were oversubscribed. However, many perceived a lack of need for instrumental support from neighbours and volunteers in food shopping during the pandemic. These individuals tended to decline the provision of support, felt they were doing fine on their own, and portrayed a desire for independence.

Before the systems were set up in position as well as they are now, originally that was always a struggle because we were relying on friends to get us our shopping. (Female, 41y, immediate family)

Participants who were considered clinically extremely vulnerable received instrumental support in the form of food parcels from the Government during the pandemic. However, all of these participants expressed a lack of desirability for these parcels. One participant of Black Caribbean ethnicity described how the food parcels did not accommodate to cultural influences on food preferences and lacked nutritional value. Nevertheless, the provision of these food parcels did make people feel emotionally supported and cared for.

[The food parcels] just made you feel like people were looking after you. (Female, 41y, immediate family)

Instrumental support appeared to play a significant role in dietary behaviour, but participants also described how informational support was important for changes to multiple health behaviours.

4. Informational support as important for behaviour change

When participants wanted to change their health behaviours during the pandemic, receiving informational support in the form of advice from family and friends was perceived to facilitate behaviour change. Receiving informational support from immediate social networks also made individuals feel emotionally supported, which was perceived as further facilitating healthy behaviours. However, many individuals were strongly disinclined to receiving advice from immediate social networks about their weight. Some described how if someone was to comment on their weight, this would cause tension in their relationship. Individuals who did not live with family and who wanted to change their health behaviours during the pandemic relied on healthcare professionals and mass media (e.g. YouTube, TV shows, books, apps) to receive lifestyle advice.

[My partner] said about getting an exercise machine, and I looked it up on Amazon and ordered it. (Male, 63y, immediate family)

Although informational support was perceived to be important for behaviour change, participants described how validation of health behaviours from immediate social networks made them feel encouraged to maintain existing behaviours.

5. Validation of health behaviours from immediate social networks

Most individuals described how family members accepted their dietary and physical activity behaviour during the pandemic, which made them feel emotionally supported. This sense of validation did not lead to changes in health behaviours but made them feel valued, supported, and encouraged. Furthermore, individuals who had lost weight during the pandemic described how receiving compliments from family and friends about their weight loss made them feel valued, encouraged a positive self-concept, and made dieting feel “worth carrying on”. However, some individuals received criticisms from family about their health behaviours during the pandemic, which made them feel less emotionally supported. Female participants described that when they received criticisms, their self-awareness increased, and they often changed their behaviours. On the other hand, male participants described that when they received criticisms, they did not change their behaviours as being criticised undermined their desire for autonomy.

[My partner] doesn’t object. She lets me get on with [exercising]. (Male, 80y, partner)

## Discussion

This study described multiple ways in which social support was perceived to impact the health behaviours of people LWBC during the COVID-19 pandemic. Support networks provided companionship and a sense of accountability for physical activity. Social norms around alcohol in the home environment strongly influenced consumption. Partners provided instrumental support in food shopping, preparation and cooking, and friends and neighbours helped people LWBC to access food whilst shielding or isolating. However, government support in the form of food parcels was not well-received due to content. When people LWBC wanted to change their health behaviours, family, friends, healthcare professionals and the media provided information and advice. Whilst validation from family and friends encouraged people LWBC to maintain existing health behaviours, criticisms from partners facilitated health behaviour change in females, but not males.

Our findings that walking with family and friends motivated people LWBC to stay physically active during the pandemic by providing an opportunity to socialise, reducing loneliness, and providing a sense of accountability are in line with a meta-synthesis of 39 qualitative studies conducted before the pandemic [19]. Furthermore, companionship support for walking was more likely to be effective when individuals’ walking abilities matched their walking partners’. This finding supports the “matching hypothesis” of social support, which posits that the effectiveness of support is greater when the support provided matches the needs of the recipient [33]. Therefore, local deliverers of physical activity interventions could run walking buddy schemes in which people LWBC who lack support in physical activity are buddied up to walk with people who live locally, and with similar abilities in walking. Furthermore, public health messaging could encourage people LWBC to walk with family and friends who have similar walking abilities to them and emphasise how this provides an opportunity to socialise yet also has many physical and mental health benefits.

Social influence from family members encouraged greater alcohol consumption in people LWBC during the pandemic. On the other hand, a lack of familial influence to drink supported lower alcohol consumption. These findings are in line with a large body of observational research conducted in the general population showing familial concordance in alcohol consumption [34, 35]. Together, these findings suggest that involving relatives in interventions designed to reduce alcohol consumption in people LWBC could help improve outcomes [36]. For example, interventions could encourage the formation of collaborative implementation intentions with family members to reduce alcohol consumption. Forming collaborative implementation intentions is a feasible way to facilitate more effective execution of implementation intentions given social influences on planning and behaviour enactment [37].

Partners of people LWBC provided instrumental support in food practices during the pandemic, especially when people LWBC lacked time, interest or skills in cooking. Contrastingly, people LWBC who felt interested and able to cook did not perceive a need for partner support. Together, these findings suggest that individuals who are already highly intrinsically motivated to cook have lower need for social support, whereas individuals who are less intrinsically motivated have greater need, in line with Self-Determination Theory [14]. The aim of the current study was not to conduct a typological analysis; however, these emerging “types” may be worth exploring in future research. Specifically, Ideal Type analysis, which has been applied previously in qualitative psychology research, could be conducted to classify people LWBC based on their feelings toward receiving social support in their health behaviours [38]. This analysis could lend itself to tailored support-based lifestyle interventions.

Instrumental support from friends and neighbours enabled people LWBC to access food during the pandemic whilst shielding, self-isolating or avoiding supermarkets. Qualitative research conducted in the general population during the COVID-19 pandemic also showed that neighbour support was important for maintaining food security [39]. However, food parcels delivered by the Government to the clinically extremely vulnerable group did not meet people’s needs for support and were said to lack nutrition. This finding supports prior reports which found that these food boxes lacked fruit and vegetable intake and were high in processed foods [40]. This is concerning given that high fruit and vegetable intake, and low processed food intake, are associated with a lower risk of overall mortality in people LWBC [8]. Future policies to deliver food parcels to clinically extremely vulnerable groups during pandemics should focus on providing healthier and more culturally appropriate food.

Informational support facilitated health behaviour change in people LWBC during the pandemic. However, informational support from immediate social networks also made people LWBC feel emotionally supported, which opposes the traditional distinction between these two types of support [31]. Other studies have also found that emotional support overlaps with informational support and have therefore questioned the distinct nature of emotional support [41–43]. Given this overlap, it is difficult to discern whether informational or emotional support had a greater impact on the health behaviours of people LWBC during the COVID-19 pandemic. Even though the types of support proposed by Cohen and Wills have been widely accepted, there is limited research investigating the value of the distinctions between these types [31, 41]. Consequently, future research should continue to explore the value of the four distinct types of social support proposed by Cohen and Wills as revisions may be necessary.

Whilst emotional support (e.g. validation) from immediate social networks encouraged the maintenance of existing health behaviours in people LWBC during the COVID-19 pandemic, negative social interactions (e.g. criticisms) facilitated health behaviour change in females, but not males. Criticisms are not considered a type of social support as they are not intended to be helpful, however, they communicate disapproval in contrast to validation which communicates acceptance. Our finding that criticisms facilitated health behaviour change in females but not males is in line with longitudinal research conducted in the general population which found that increased relationship strain was associated with greater decreases in alcohol consumption over time in females, but not males [44]. It is currently unclear why these sex differences exist. One possible explanation is that females have greater social networks than males, and thus receive positive support from social relations outside their marital relationship, which buffers them from the potential negative effects of social control [44, 45]. Future qualitative research could continue to explore why males and females LWBC react differently to social control, as this could lend itself to sex-specific couple-based lifestyle interventions. Nevertheless, the findings from this study suggest that partners could be integrated into lifestyle interventions for people LWBC, and that supportive communication could be encouraged between them. For example, couple-based communication workshops could teach partners how to communicate with each other about health behaviours in a more supportive way.

### Strengths and limitations

This study shows how social support was perceived to impact the health behaviours of people LWBC during the COVID-19 pandemic. This knowledge is particularly important for designing support-based lifestyle interventions [46]. Furthermore, using the theory of types of social support proposed by Cohen and Wills facilitated a greater understanding of the types of support that impacted their health behaviours, thus enabling more specific recommendations for support-based lifestyle interventions [31]. However, some limitations of the study include data collection at a single time point which prevents an in-depth understanding of how the perceived impact of social support on health behaviours changed during the COVID-19 pandemic amidst ever-changing social restriction measures. Although every attempt was made to purposefully sample a diverse range of people LWBC, most participants were White British, married/cohabiting with a partner, and living in urban areas, which limits the transferability of the findings. Participants had also chosen to participate in a trial of a lifestyle intervention, and some had received this intervention, and so might be more health conscious than the general population.

### Conclusions

This study has described how companionship, social influence, instrumental support, informational support and validation were perceived to impact the health behaviours of people LWBC during the COVID-19 pandemic. Future lifestyle interventions for people LWBC could involve their relatives and/or friends and promote the supportive behaviours identified in this study. Such interventions would be useful in pandemics and in a non-pandemic environment and could increase engagement in health behaviours in people LWBC. Furthermore, government policies to help clinically extremely vulnerable groups access food during pandemics should focus on providing access to healthier foods, as this has implications for the outcomes of people LWBC.

## Supplementary Information

Below is the link to the electronic supplementary material.

Supplementary file1 (DOCX 20 KB)

## Data Availability

The data are not available because the transcripts contain sensitive information.

## Code availability

N/A.
