# Supplementary material for: Exploring the perceived impact of social support on the health behaviours of people living with and beyond cancer during the COVID-19 pandemic: a qualitative study
Source: Support Care Cancer. 2022 Jul 25;30(10):8357–66. doi: 10.1007/s00520-022-07291-0 (PMC9311339; doi:10.1007/s00520-022-07291-0)
Supplement: Supplementary file 1 — Supplementary file1 (DOCX 20 KB) [file 520_2022_7291_MOESM1_ESM.docx]

**Topic guide**

**Introduction**

- *Introduce self. I am a researcher on behalf of the ASCOT trial.*
- *Thank participant for agreeing to participate in the interview. Tell them there are no right/wrong answers.*
- *Ask for permission to record. Remind them to not use names in order to maintain anonymity. Start recording.*
- Thank you for filling in the survey about your experiences during the COVID-19 pandemic and for agreeing to be interviewed. We are really interested in how people have experienced the pandemic and especially whether family, friends or other people have affected things like what you eat or how much physical activity you do.
- To start with, it would be good if we could begin with some general questions.

**Diagnosis**

Can you tell me a bit about your cancer diagnosis?

**Daily life**

Can you tell me how you are spending your days at the moment?

- Prompts: *Do you work? Are you working from home?*

How has this changed from before COVID-19?

**Living arrangements**

Can you tell me a bit about your current living arrangements?

- *Do you live with anyone or are you living alone?*

How has this changed from before COVID-19?

**Physical environment**

Can you tell me a bit about the area where you live?

- *Do you live near shops/supermarkets, restaurants/bars, outdoor green spaces/gyms?*
- *Do you have a garden?*

Do you live near friends/family?

**Social support in diet**

How would you describe what you currently eat?

How has this changed from before COVID-19?

- *How many meals do you eat per day?*
- *Do you follow any particular diet?*
- *Can you tell me about your setup at home in terms of food shopping, meal planning, cooking, eating?*
- *Do you eat the same foods as your family/household?*
- *How do you feel about your partner/family/friends planning your food shopping/planning your meals/cooking for you/eating with you?*
- *What does your partner/family/friends think of what you eat?*

**Social support in physical activity**

How much physical activity are you doing at the moment?

How has this changed from before COVID-19?

- *What types of exercise do you do?*
- *How often do you exercise?*
- *Do you exercise with anyone?*
- *If exercise with others: Can you tell me a bit about why you exercise with your partner/family/friends? How do you feel about exercising with your partner/family/friends?*
- *Can you tell me about your partner’s/family’s/friends’ physical activity behaviour?*
- *Do you ever discuss your exercise behaviour with your partner/family/friends?*
- *What does your partner/family/friends think about your exercise behaviour?*

**Social support in weight management**

How do you find managing your weight at the moment?

How has the pandemic affected your ability to manage your weight?

- *Can you tell me about how your partner/family/friends help you to manage your weight? Do your partner/family/friends provide any encouragement or support?*
- *If yes: How does the support help you manage your weight? How do you feel about the support they provide?*
- *What does your partner/family/friends think about your ability to manage your weight?*
- *Do you attend any weight loss programmes e.g., weight watchers, slimming world?*
- *If yes: why do you attend these programmes? How do you feel about them?*

**Social support in alcohol consumption**

Can you tell me about your current drinking behaviour?

How has this changed from before COVID-19?

- *How often do you drink?*
- *When do you drink?*
- *What are your reasons for drinking/not drinking?*
- *Do you drink with anyone?*
- *If drink with others: Can you tell me a bit about why you drink alcohol with your partner/family/friends? How do you feel about drinking alcohol with your partner/family/friends?*
- *Can you tell me about your partner’s/family’s/friends’ drinking behaviour?*
- *What does your partner/family/friends think about your drinking behaviour?*

**Conclusion**

- Is there anything that we have not discussed today about support received from family, friends or other people during the COVID-19 pandemic that you would like to tell us about?
- Do you have any questions?
- Thank you for your time and participation today!

**Transition between initial frameworks to final themes**

**Framework 1**

*Deductive coding: Cohen and Will’s (1985) four types of social support*

1. Social Companionship
2. Instrumental Support
3. Informational Support
4. Emotional Support

**Framework 2**

*Framework integrating inductive and deductive codes*

1. Companionship:
2. Opportunities to socialise
3. Feelings of safety
4. Accountability
5. Mismatch between companionship support received and desired
6. Instrumental support:
7. Access to food
8. Maintenance of eating habits
9. Encouragement to exercise
10. Helping people to cut down alcohol consumption
11. Informational support:
12. Suggestions from informal social networks
13. Advice from formal relations
14. Emotional support:
15. Validation
16. Compliments and encouragement
17. Criticisms

**Framework 3**

*Framework after double coding and amendments*

1. Companionship:
2. Co-participation in exercise
3. Social drinking
4. Instrumental support in food practices:
5. Access to food when shielding/isolating/avoiding supermarkets
6. Partner support
7. Informational support:
8. Suggestions from informal social networks
9. Advice from formal relations
10. Emotional support:
11. Validation

**Framework 4**

*Final framework*

1. Companionship and accountability as motivators for physical activity
2. Social influences on alcohol consumption
3. Instrumental support in food practices
4. Informational support as important for behaviour change
5. Validation of health behaviours from immediate social networks
